# Developmental epigenetic circuits and their epigenetic drift in skeletal muscle ageing

**DOI:** 10.1098/rstb.2025.0342

**Published:** 2026-11-12

**Authors:** Laurent Schaeffer, Liudmila Afanaseva, Edwige Belotti

**Affiliations:** ^1^ Université Claude Bernard Lyon 1, Laboratory of Pathophysiology and Genetics of Neuron and Muscle (PGNM), Institut NeuroMyoGène, INSERM U1315, CNRS UMR5261, 8 avenue Rockefeller, 69008 Lyon, France; ^2^ Centre de Biotechnologie Cellulaire, Hospices Civils de Lyon, Lyon, France

**Keywords:** skeletal muscle, ageing, epigenetics, sarcopenia, genome integrity, long-lived cells

## Abstract

Development and ageing are two deeply intertwined processes, but it remains unclear how programmes established during early development influence the long-term stability of post-mitotic tissues. Skeletal muscle provides a model of choice to study the connection between these processes. Myofibres are long-lived, multinucleated syncytia whose identity and homeostasis rely on transcriptional, metabolic and epigenetic states established during embryonic and postnatal development. Throughout life, these states are further reinforced by satellite cell-derived inputs. In this review, we discuss how early specification cues, chromatin architecture and metabolic wiring establish durable regulatory circuits preserving myofibre integrity but also limiting its plasticity across the lifespan. These developmental features, combined with decades of physiological strain, progressively challenge genomic integrity and destabilize the maintenance of epigenetic information. Over time, this instability causes an epigenetic drift, encompassing alterations in DNA methylation, changes in histone landscapes and disruption in higher-order chromatin organization. Such alterations erode transcriptional fidelity and contribute to the appearance of hallmarks of the ageing phenotype. By integrating developmental and epigenetic perspectives, we argue that myofibre ageing reflects not only cumulative damage but also the long-term consequences of how these exceptionally long-lived cells were originally built.

This article is part of the theme issue ‘Developmental circuits in ageing’.

## 1. Introduction

Ageing manifests as a progressive decline in physiological functions that compromises homeostasis, regenerative capacity and stress resilience, thereby increasing susceptibility to chronic diseases. Skeletal muscle, the largest organ system of the human body, comprising more than 600 muscles and representing approximately 40% of the body mass in healthy young adults, is essential for locomotion, energy homeostasis and thermoregulation [1,2]. Sarcopenia, the age-related loss of muscle mass, strength and function, impairs mobility, elevates the risk of falls and fractures, reduces autonomy and enhances comorbidities, imposing major socioeconomic and healthcare burdens [3–5].

The contractile cells of skeletal muscle are multinucleated myofibres, among the largest cells in the body. Muscle fibres result from the fusion of mononucleated precursor cells during embryonic development and postnatal growth. Some of these progenitors become quiescent and lie along myofibres under the basal lamina (hence their name, satellite cells) throughout their lives. They are reactivated upon muscle injuries to proliferate and regenerate new muscle fibres. After strenuous physical exercise, they can also contribute to reinforcing damaged myofibres [6,7]. Myofibres exhibit remarkable structural specialization, including peripheral nuclear positioning, highly ordered contractile myofibrillar arrays and extensive intra-cellular transport networks, to enable efficient contraction and molecular distribution across a vast cytoplasm [1,2]. Satellite cells constitute the postnatal pool of muscle stem cells (MuSCs).

Skeletal muscle is the main reserve of amino acids in the organism and a major consumer of oxygen and nutrients. To avoid unnecessary energy expenditure and ensure optimal function, skeletal muscles show remarkable plasticity to adapt their metabolic (glycolytic or oxidative), contractile properties (fast or slow) and size to the demand and environmental conditions. As a result, malnutrition, hypoxia (high altitude) and inactivity cause muscle atrophy to reduce its size, hence its consumption of oxygen and nutrients. Conversely, exercise training, such as weight lifting, increases muscle mass and favours powerful fast glycolytic fibres, whereas endurance training favours oxidative fibres [8–10]. However, such plasticity also imposes vulnerabilities that become exacerbated with ageing and in pathologies. Hallmarks of muscular ageing include muscle atrophy, with a preferential atrophy of fast glycolytic fibres, alteration in myonuclear organization, progressive impairment of satellite-cell function and gradual decline of the contractile function [1,2,11–13]. These age-related changes are part of the 12 hallmarks of ageing, which highlight a set of interconnected biological processes, grouped into primary, antagonistic and integrative hallmarks that collectively shape ageing trajectories [14]. Many of these hallmarks, including mitochondrial dysfunction, loss of proteostasis, deregulated nutrient sensing and cellular senescence, are strongly represented in skeletal muscle. Additional muscle-specific processes, such as the progressive deterioration of neuromuscular junctions (NMJs), further underscore the complexity of sarcopenia [15]. Despite this mechanistic diversity, a unifying theme has emerged: multiple primary and integrative ageing hallmarks converge on nuclear dysfunction as a central driver of muscular ageing.

In skeletal muscle, nuclear integrity is challenged lifelong by high metabolic demand, high mechanical strain and the extensive transcriptional activity required to maintain NMJs and sustain large syncytial cells. With age, these pressures cause many age-associated alterations, including DNA damage accumulation, impaired DNA-repair pathways, changes in coding and non-coding RNA expression, alterations in nuclear envelope architecture, telomere shortening and extensive epigenetic remodelling [16–18]. Epigenetic mechanisms, including DNA methylation, histone modifications, nucleosome positioning, histone variant turnover and higher-order chromatin organization, constitute a fundamental layer of genomic regulation that preserves cellular identity while remaining vulnerable to cumulative stress [19]. Recent conceptual models, including the ‘Information Theory of Ageing’, suggest that the progressive erosion of epigenetic information, through chromatin disorganization, altered histone landscapes and increased transcriptional noise, acts as a reversible driver of ageing [20]. In long-lived post-mitotic cells, such as myofibres and differentiated neurons, the requirement to preserve chromatin integrity for decades amplifies the consequences of epigenetic instability.

In this review, we examine the developmental origins and epigenetic basis of skeletal muscle ageing. An emerging concept is that chromatin states, established during myogenesis, form long-lasting regulatory circuits that support adult muscle physiology but progressively constrain its adaptability with age. Their disruption contributes to impaired regeneration, altered fibre identity and functional decline. By considering muscle ageing in a lifespan perspective, from developmental programming to late-life degeneration, this review proposes a mechanistic framework for understanding sarcopenia and highlights epigenetic processes as potential targets for therapeutic rejuvenation.

## 2. Part 1A: developmental origins of epigenetic memory in the muscle lineage

### (a) Embryonic myogenesis and satellite cell specification

Skeletal muscle development provides a powerful framework for understanding how early gene regulatory programmes establish chromatin states that persist throughout the lifespan. In vertebrates, skeletal muscles arise from paraxial mesoderm progenitors that commit to the myogenic lineage, proliferate as myoblasts and become myocytes that ultimately fuse to form multinucleated myofibres. In mice, myogenesis proceeds through two temporally and functionally distinct waves that shape muscle architecture and cellular diversity [21–23].

Primary (embryonic) myogenesis occurs approximately between embryonic day E9.5 and E11 and establishes the initial muscle pattern through the fusion of embryonic mononucleated myocytes into nascent primary myofibres. Secondary (fetal) myogenesis, occurring between E14.5 and E17.5, expands muscle mass by generating the majority of definitive fibres through fusion of fetal myocytes either *de novo* or along the primary fibre scaffold [23].

These successive developmental waves expose myogenic progenitors to dynamic signalling environments and chromatin states that prefigure long-term epigenetic heterogeneity. During these transitions, Pax3 is highly expressed in early embryonic progenitors, whereas Pax7 expression becomes progressively enriched in fetal progenitors and marks the population that will give rise to satellite cells [21,24]. Beyond their canonical transcriptional roles, Pax transcription factors are thought to contribute to the establishment of lineage-restricted chromatin accessibility, thereby stabilizing a form of epigenetic memory within muscle [25–27].

At the end of fetal myogenesis, a subset of fetal progenitors withdraws from the differentiation programme and becomes specified as long-term MuSCs (satellite cells), which localize beneath the basal lamina and are maintained throughout life [21]. The chromatin configurations established during fetal development are inherited by postnatal satellite cells and repeatedly redeployed during adult regeneration.

### (b) Adult muscle regeneration as a redeployment of developmental programmes

While embryonic and fetal myogenesis establish the foundational architecture of skeletal muscle, a defining feature of this tissue is its lifelong regenerative capacity. This process is driven by satellite cells, the resident MuSCs specified during late fetal development. Rather than constituting a *de novo* programme, adult muscle regeneration relies on the reactivation of developmental gene regulatory networks [24,25,28].

Upon muscle injury, quiescent MuSCs exit dormancy, re-enter the cell cycle and give rise to proliferating myoblasts that differentiate and fuse to restore damaged myofibres [28]. This process recapitulates key aspects of developmental myogenesis, including the sequential activation of myogenic regulatory factors (MRFs) and the re-engagement of major signalling pathways such as Wnt, Notch, BMP and TGF-β [24,29].

At the chromatin level, regeneration is facilitated by the persistence of permissive and poised epigenetic configurations established during development, enabling rapid and coordinated transcriptional responses upon activation [30]. The underlying mechanisms are discussed in the following sections. Importantly, regeneration occurs in a distinct physiological context compared with embryonic development. It is shaped by systemic and local cues, such as inflammation, metabolic status and niche-derived signals [29,31,32].

Thus, adult muscle regeneration can be viewed as a reiterative deployment of developmental epigenetic programmes operating within a more complex and progressively altered environment. This framework provides a basis for understanding how these programmes both sustain tissue repair and contribute to age-associated functional decline when their regulation becomes compromised.

### (c) Epigenetic control of myogenic progression

#### (i) Polycomb complexes and bivalent chromatin state during myogenic commitment

Embryonic and adult myogenic precursors and their progeny are subjected to tight epigenetic regulation throughout myogenesis. MRFs, including Myf5, MyoD, myogenin and MRF4, together with Myocyte Enhancer Factor 2 (MEF2) transcription factors, coordinate lineage commitment, proliferation and terminal differentiation. Their activity is tightly controlled by chromatin modifiers, such as histone acetyltransferases (HATs) and deacetylases, histone methyltransferases and demethylases and ATP-dependent remodelling complexes, including SWI/SNF, which dynamically regulate accessibility to promoters and enhancers of muscle genes [33,34].

In proliferating myoblasts, many developmental genes remain transcriptionally poised. This state, characterized by bivalent chromatin domains marked by the concomitant presence of the active mark H3K4me3 and of the repressive mark H3K27me3, is accompanied by additional repressive histone modifications. Polycomb repressive complexes (PRCs) play a central role in establishing and maintaining these chromatin states. PRC2, whose catalytic component EZH2 deposits H3K27me3, contributes to the repression of differentiation-associated genes, whereas PRC1 complexes, including BMI1-containing variants, reinforce repression through monoubiquitination of histone H2A. In parallel, the histone methyltransferase SUV39H1 deposits H3K9me3 to stabilize gene repression at multiple stages of myogenesis, including the silencing of S-phase and non-muscle genes, thereby preventing cell cycle re-entry of differentiating myoblasts and contributing to the maintenance of satellite cell quiescence [33–35].

These repressive chromatin configurations remain dynamically regulated during adult muscle regeneration. Upon satellite cell activation, PRC2-dependent H3K27me3 transiently restrains differentiation genes during early activation. This repression is resolved through p38α-dependent phosphorylation of EZH2 at threonine 372, promoting Praja1/PJA1-mediated ubiquitination and proteasomal degradation of EZH2, thereby enabling timely derepression of myogenic programmes [36]. The p38α signalling also drives phosphorylation-dependent recruitment of YY1–PRC2 to the Pax7 locus, reinforcing repression of self-renewal genes during commitment [37].

#### (ii) Histone variant deposition and myogenic plasticity

In addition to canonical histone modifications, replication-independent histone variant deposition provides an additional and critical layer of chromatin diversity during myogenesis.

Among these, the histone variant H3.3, deposited by the HIRA and DAXX/ATRX chaperone systems, is enriched at myogenic promoters and enhancers where it promotes increased nucleosome turnover and chromatin accessibility. H3.3 enrichment is frequently associated with H3K27ac deposition and facilitates rapid transcriptional activation during differentiation. At the same time, it remains compatible with the maintenance of bivalent H3K4me3/H3K27me3 domains, thereby preserving developmental plasticity [27,38].

Mechanistically, MEF2 transcription factors actively promote HIRA-mediated replacement of canonical H3.1 by H3.3 at myogenic loci. This histone exchange relieves PRC2-dependent H3K27me3 repression and enables efficient myoblast differentiation. Conversely, incorporation of H3.1 in proliferating progenitors favours PRC2 recruitment, stabilizes repressive chromatin states and impairs myogenic progression, underscoring the instructive role of histone variant choice in lineage commitment [39,40].

Beyond HIRA-mediated deposition, the DAXX/ATRX complex, classically associated with heterochromatin regulation, also contributes to myogenic transcription through H3.3 incorporation at a distinct subset of loci. *Daxx*-deficient myoblasts exhibit impaired differentiation and defective fusion accompanied by reduced H3.3 and H3K27ac enrichment at key myogenic genes, such as *MyoD*. These findings indicate that DAXX-mediated H3.3 deposition acts through mechanisms distinct and complementary to the HIRA-dependent pathway, highlighting functional specialization among H3.3 chaperone systems during muscle differentiation [41].

In parallel, histone H2A variants provide complementary regulatory inputs at myogenic regulatory elements. Acetylation of H2A.Z at the *MyoD* transcriptional start sites promotes an open chromatin configuration and facilitates transcription factor recruitment, whereas expression of non-acetylatable H2A.Z mutants prevents *MyoD* induction and blocks myoblast differentiation [42]. The SRCAP chromatin remodelling complex further deposits H2A.Z at the *Myogenin* (*Myog*) promoter in a p38 MAPK-dependent manner. Phosphorylation of the SRCAP subunit ZNHIT1 (p18^Hamlet^) by p38 MAPK is required for H2A.Z incorporation, which in turn enables transcriptional activation of *Myogenin* and progression of the differentiation programme. Consistently, downregulation of SRCAP subunits disrupts this H2A.Z-mediated chromatin remodelling, identifying the SRCAP–p38–H2A.Z axis as an early regulatory module controlling myogenic differentiation [43].

Another histone variant, MacroH2A1.2, accumulates selectively at muscle metabolic enhancers during differentiation, where it recruits the transcription factor Pbx1 and potentiates MyoD-dependent activation of oxidative and slow-fibre gene programmes, thereby contributing to metabolic specialization and fibre-type regulatory networks [44]. Distinct MacroH2A1 splice variants exert divergent functions: MacroH2A1.1 promotes myoblast fusion through the upregulation of extracellular matrix and adhesion genes, whereas MacroH2A1.2 inhibits fusion. This differential splicing provides an additional regulatory layer which was proposed to be disrupted in aged satellite cells [45].

Together, these replication-independent histone variants (H3.3 deposited by HIRA and DAXX/ATRX, H2A.Z and MacroH2A1 isoforms) establish a dynamic chromatin regulatory layer superimposed on Polycomb- and Trithorax-controlled chromatin landscapes. This multi-layered epigenetic architecture balances transcriptional restraint in embryonic progenitors with rapid gene activation during differentiation, while preconfiguring chromatin states that will later be reused by postnatal satellite cells. For an exhaustive overview of histone variant function in myogenesis, we refer the reader to recent detailed reviews [46,47].

#### (iii) Diverse functions of histone demethylases in myogenesis

In parallel with Polycomb-mediated repression and histone variant deposition, histone demethylases provide an essential mechanism contributing to the reversibility and temporal control of chromatin states during myogenesis. Members of the histone lysine demethylase (KDM) family, including FAD-dependent enzymes, such as lysine-specific demethylase 1 (LSD1/KDM1A) and Fe(II)/α-ketoglutarate (α-KG)-dependent JmjC domain-containing demethylases, dynamically remove methyl groups from histone lysine residues, thereby fine-tuning transcriptional programmes that regulate myogenic commitment, differentiation and muscle stem cell fate decisions [48].

Among these enzymes, LSD1/KDM1A has emerged as a key regulator of skeletal muscle differentiation. Loss of LSD1 impairs C2C12 myoblast differentiation and disrupts timely expression of *MyoD* during limb development in E11.5 mouse embryos through regulation of the *MyoD* core enhancer [49,50]. Mechanistically, LSD1 interacts with MyoD and MEF2D to remove the repressive H3K9me2 mark from promoters of differentiation-associated genes such as *Myogenin* and muscle creatine kinase (*MCK*), thereby facilitating transcriptional activation [49,51].

LSD1 also contributes to early myogenesis via the demethylation of non-histone targets. Demethylation of β-catenin by LSD1 prevents its degradation upon nuclear entry, thereby enabling the Wnt/β-catenin pathway to control MuSC asymmetric division and the activation of the *MyoD* core enhancer [50,52].

Other demethylases similarly contribute to the removal of repressive chromatin marks at myogenic loci. KDM4C/JMJD2C interacts with MyoD to remove the repressive H3K9me3 mark at myogenic promoters, while simultaneously blocking MyoD methylation by G9a, thereby preventing its degradation. Through this dual mechanism, KDM4C stabilizes *MyoD* transcriptional activity during differentiation [53]. In parallel, KDM6A/UTX dynamically removes Polycomb-dependent H3K27me3 marks at muscle-specific genes during myogenesis. UTX is initially recruited to regulatory regions by transcriptional activators, such as Six4, after which RNA polymerase II-mediated elongation facilitates H3K27me3 removal across gene bodies, establishing transcriptionally permissive chromatin regions [54].

Overall, histone demethylases provide a dynamic counterbalance to repressive chromatin states during myogenesis. For a more detailed explanation of histone demethylase regulatory pathways, we refer the reader to a recent review [48].

Altogether, Polycomb-mediated repression, bivalent chromatin domains, histone variant deposition and histone demethylation establish a multi-layered epigenetic architecture during development, providing reversibility to transcriptional repression to allow future responsiveness. These chromatin configurations are inherited by postnatal satellite cells, where they support quiescence while allowing efficient activation and regeneration upon injury. For a broader overview of histone modification regulators in skeletal muscle development, regeneration and disease, we refer the reader to a recent review [55].

## 3. Part 1B: redeployment of epigenetic memory in adult regeneration

### (a) Dynamic quiescence–activation epigenetic transitions

In postnatal skeletal muscle, MuSCs or Pax7⁺ satellite cells reside in a specialized niche between the basal lamina and sarcolemma, where they maintain a reversible quiescent G_0_ state. Quiescence is not a passive condition but an actively maintained epigenetic state characterized by low transcriptional and metabolic activity that preserves regenerative potential and enables rapid reactivation upon injury [32,56–58]. Importantly, this state reuses chromatin configurations established during embryonic and fetal myogenesis. Ultrastructurally, quiescent MuSCs display nuclei enriched in compact heterochromatin. At the molecular level, they retain bivalent chromatin domains at lineage-specific loci, reflecting a poised epigenetic configuration reminiscent of embryonic progenitors and other adult stem cells [57,59–61].

Maintenance of quiescence depends on precise heterochromatin organization. The H4K20 methyltransferase SUV4-20H1 deposits H4K20me2/3 at the *MyoD* locus, promoting facultative heterochromatin that prevents premature differentiation while preserving accessibility at *Pax7* regulatory regions required for stem cell identity and asymmetric division [62]. Polycomb complexes further refine this balance. The PRC2 paralog EZH1 restrains spontaneous activation by maintaining transcriptional programmes associated with quiescence, including Notch signalling, whereas EZH2 becomes required during activation and early proliferative expansion. Loss of EZH1 leads to inappropriate MuSC activation, depletion of the Pax7⁺ pool and defective regeneration, highlighting the complementary roles of Polycomb paralogs in adult stem cell maintenance [63].

Functional heterogeneity within the MuSC pool reflects graded epigenetic states. Cells with high levels of Pax7 expression (Pax7^Hi^) exhibit deep quiescence, reduced metabolic activity and delayed entry into the cell cycle, whereas cells with low levels of Pax7 expression (Pax7^Low^) are primed for rapid activation. This continuum of states among satellite cells enables flexible responses to niche-derived cues while preserving long-term self-renewal capacity, a property rooted in developmental chromatin programming [56].

Upon injury, MuSCs exit quiescence and activate the myogenic transcriptional programme, including the reactivation of the MRFs, MEF2 family members and Pax/Six transcription factors, together with developmental signalling pathways, such as Wnt, Shh, BMP and TGF-β. These pathways induce rapid remodelling of chromatin landscapes by HATs, histone deacetylases (HDACs), histone methyltransferases and demethylases, as well as ATP-dependent chromatin-remodelling complexes, such as SWI/SNF [33,34,64–66]. Distribution of histone variants initially established during embryogenesis is also dynamically redeployed, facilitating rapid gene induction and contributing to fibre-type specification during regeneration [46]. Thus, adult muscle regeneration does not recapitulate development *de novo,* but instead reuses preconfigured epigenetic programmes established during embryogenesis to enable efficient and timely repair.

### (b) Metabolic-epigenetic coupling

Muscle stem cell identity relies on tight coupling between metabolism and epigenetic programmes that govern quiescence, activation and self-renewal. In young MuSCs, DNA methylation and histone modification dynamics follow highly ordered trajectories that depend on the availability of metabolic co-factors, such as S-adenosylmethionine (SAM), NAD⁺ and α-KG [67]. The epigenetic programmes redeployed during muscle regeneration are therefore tightly coupled to the metabolic state of MuSCs. This coupling links nutrient availability and energy flux to chromatin remodelling and transcriptional control, ensuring precise activation and differentiation. In quiescent MuSCs, reliance on fatty acid oxidation and oxidative phosphorylation (OXPHOS) sustains a high NAD⁺/NADH ratio, which promotes the activity of the NAD⁺-dependent deacetylase SIRT1. The sirtuin-family member SIRT1 deacetylates H4K16 and MyoD, restraining myogenic gene expression and stabilizing the quiescent state [68]. Following injury, MuSCs rapidly shift towards a glycolytic metabolism, reducing NAD⁺ availability, thereby attenuating SIRT1 activity and promoting H4K16 acetylation. Simultaneously, acetyl-CoA produced by pyruvate dehydrogenase (PDH) and the tricarboxylic acid (TCA) cycle fuels global histone acetylation (H3K9ac, H4K16ac and H2BK5ac) at promoters of cell cycle and stemness genes during early activation, before being redirected to *MyoD* and *Myog* regulatory regions to drive myogenic commitment and myotube growth [69,70]. Similarly, Pax7 acetylation depends on acetyl-CoA availability and SIRT2 deacetylase activity. The balance between acetylation by MYST1 and deacetylation by SIRT2 modulates Pax7 activity. Consistently, loss of Pax7 acetylation alters satellite cell division outcomes and fibre type specification by shifting the balance between symmetric self‑renewing and asymmetric differentiating divisions, thereby modifying the proportion of self‑renewing satellite stem cells and their differentiation potential [71].

In addition to sirtuins, poly(ADP-ribose) polymerases (PARPs) have emerged as key NAD⁺-dependent actors of the metabolic–epigenetic interface. PARP1 consumes NAD⁺ to catalyse poly(ADP-ribosyl)ation (PARylation) of histones and chromatin-associated proteins, thereby modulating chromatin accessibility and transcriptional responses. Because PARP1 and SIRT1 compete for the same NAD⁺ pool, fluctuations in NAD⁺ during MuSC activation can shift the balance between deacetylation and ADP-ribosylation, fine-tuning chromatin states [72]. PARP1 restrains differentiation by limiting MyoD-dependent gene activation. It associates with MyoD-bound loci and correlates with loss of the activating mark H3K4me3. PARP1 expression decreases during differentiation, and its depletion enhances induction of MyoD targets, such as *Myog*, *Mef2c*, *p21* and *p57* [73]. By contrast, PARP3 promotes myogenesis by facilitating deposition of the repressive mark H3K27me3 on selected genes, enhancing EZH2 recruitment and ADP-ribosylating EZH2 to fine-tune PRC2-mediated repression [74]. Together, PARP family members act as metabolic sensors that integrate NAD⁺ availability with chromatin remodelling to orchestrate precise myogenic progression.

One-carbon metabolism and α-KG further link nutrient sensing to chromatin remodelling. Activation-induced upregulation of the serine synthesis pathway increases the production of SAM, the universal methyl donor required for DNA and histone methylation [75]. SAM supports EZH2-mediated H3K27 methylation at MuSC self-renewal associated loci, such as *Pax7* [37,76], and more broadly sustains the activity of MuSC-relevant methyltransferases, including the DNA methyltransferase Dnmt3a to promote proliferation of myogenic progenitors [77], the histone lysine methyltransferase SUV4-20H1 to maintain quiescence [62] and the arginine methyltransferase Carm1 (Prmt4), which methylates *Pax7* to recruit the MLL1/2 H3K4 methyltransferase complex and activate *Myf5* transcription [78]. Concurrently, α-KG serves as a co-factor for Jumonji C (JmjC) domain histone demethylases and for TET proteins involved in DNA demethylation [55,79]. Whether fluctuations in α-KG quantitatively modulate the activity of the H3K27 demethylase JMJD3 during MuSC activation remains to be fully established. These opposing methylation and demethylation reactions dynamically tune lineage progression and self-renewal [80].

Besides one-carbon metabolism and α-KG, the arginine methyltransferase Carm1 provides an additional layer of metabolic–epigenetic integration. By coordinating local methylation landscapes in response to cellular redox and energy states, Carm1 promotes transcriptional activation of myogenic genes [81]. Carm1 activity and subcellular localization are in turn tightly regulated by these states. In the presence of oxidative stress, p38γ MAPK phosphorylates Carm1 at S595, promoting its cytoplasmic retention and thereby limiting its action on Pax7 [82,83]. Cytoplasmic Carm1 instead targets proteins involved in mitochondrial dynamics, such as the mitochondrial fission factor DRP1 [84].

While the redeployment of developmental epigenetic memory enables efficient regeneration in young muscle, ageing progressively destabilizes these finely tuned chromatin–metabolic circuits. Disruption of NAD⁺, SAM and α-KG flux, combined with altered enzyme localization and mitochondrial dysfunction, impairs quiescence control, transcriptional fidelity and regenerative potential. As will be detailed in the following sections, these age-associated perturbations underpin metabolic rewiring, epigenetic drift and chromatin rigidity in aged MuSCs, converting adaptive developmental circuits into pathological constraints.

## 4. Part 2: muscle stem cells ageing

Ageing imposes progressive intrinsic and extrinsic alterations on MuSCs, leading to decline in stem cell number, impaired quiescence control, defective self-renewal and diminished regenerative capacity, thereby contributing to sarcopenia [85,86]. Rather than declining uniformly, the MuSC pool becomes increasingly heterogeneous with age, developing different subsets displaying chronic activation, delayed cell cycle entry, premature differentiation or failed activation (figure 1). This heterogeneity is associated with altered transcriptional kinetics and an impaired capacity to sustain coordinated muscle repair [87–89]. Altogether, rather than simply attenuating stem cell potential, ageing disrupts the integrity and coordination of the regulatory circuits established during development.

**Figure 1.**
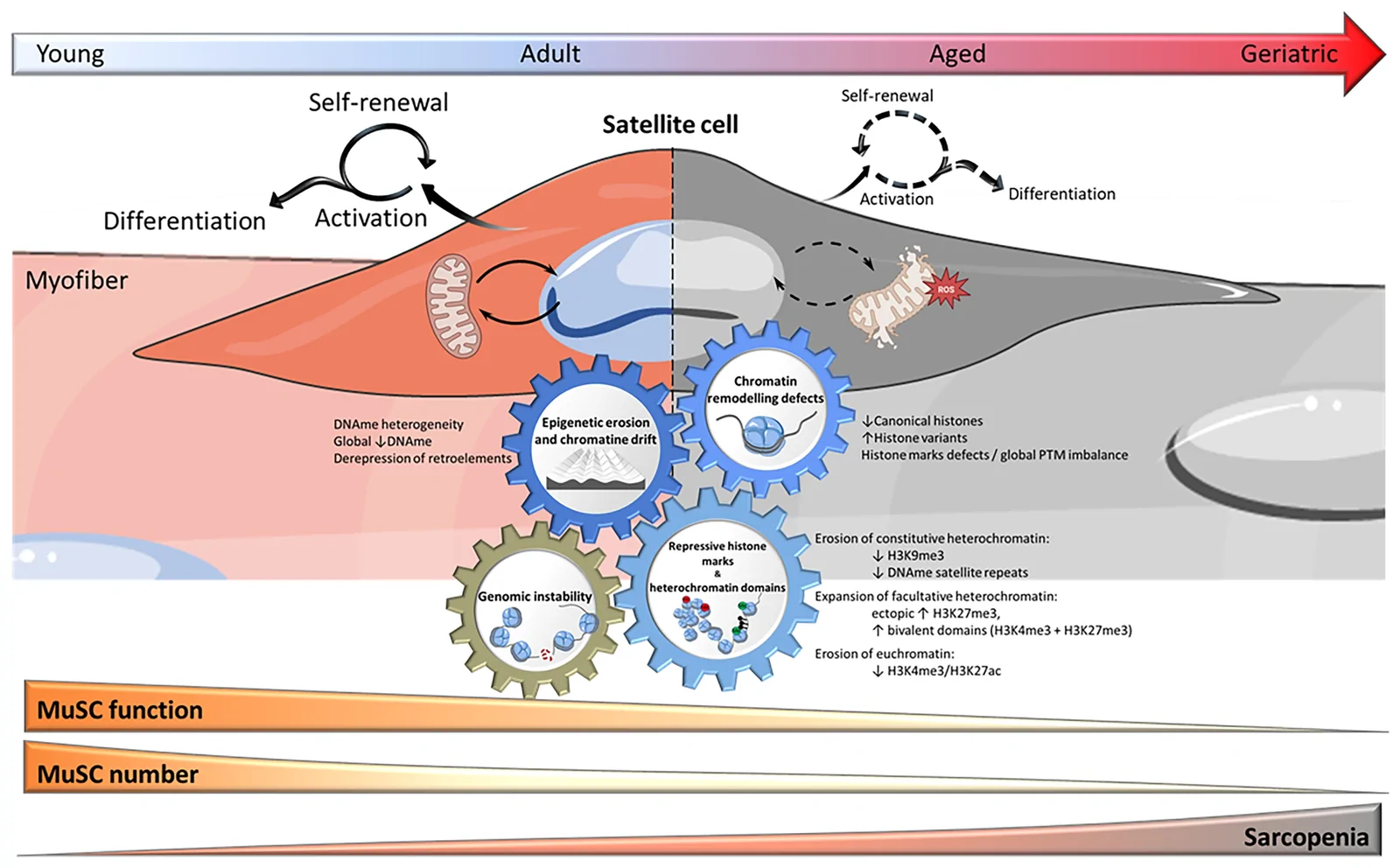
Schematic representation of chronological epigenetic alterations in MuSC nuclei during ageing. In young MuSCs, active maintenance of chromatin state enables myogenic programme redeployment upon activation and efficient return to quiescence. In aged MuSCs, epigenetic drift progressively erodes this epigenetic control, compromising quiescence stability, self-renewal and regenerative capacities. This multi-layered chromatin drift manifests at several levels: epigenetic erosion and chromatin drift, chromatin remodelling defects, heterochromatin domain reorganization with erosion of constitutive heterochromatin, ectopic expansion of facultative heterochromatin and euchromatin erosion at specific loci. These regulations are tightly coupled to the metabolic state of the cell and nucleo-mitochondrial communication, which progressively destabilizes, diminishing the supply of co-factors (NAD⁺, acetyl-CoA and SAM) required by chromatin-modifying enzymes. This progressive mitochondrial dysfunction exacerbates these effects through generation of oxidative stress. Accumulated reactive oxygen species (ROS) further drives somatic mutations and DNA lesions, establishing a vicious feed-forward loop of genomic instability that locks aged MuSCs in epigenetic dysfunction. Ultimately, this progressive epigenetic dysfunction reduces MuSC number and regenerative potential, directly contributing to sarcopenia.

### (a) Chromatin and DNA methylation drift in aged muscle stem cells

At the chromatin level, ageing manifests as progressive epigenetic imprecision rather than uniform repression or activation. Quiescent aged MuSCs undergo extensive heterochromatin reorganization, characterized by increased deposition and redistribution of H3K27me3 across promoters, intergenic regions and bivalent loci [57]. Notably, histone gene clusters marked by H3K4me3 alone in young quiescent MuSCs become aberrantly bivalent with age, accompanied by reduced histone gene expression.

By contrast, in activated MuSCs, where H3K27me3 expansion correlates with transcriptional changes involving *Ezh2* and *Jmjd3*, aged quiescent MuSCs do not display major alterations in the expression of these enzymes. This suggests that H3K27me3 accumulation may reflect altered Polycomb distribution or targeting rather than transcriptional regulation of *Ezh2* or *Jmjd3* [57]. Beyond a global increase in H3K27me3, aged MuSCs stochastically accumulate bivalent chromatin regions at core histone gene clusters and developmental loci, accompanied by locus-specific gains and losses of the activation mark H3K4me3. This pattern reflects chromatin drift rather than coordinated repression, indicating progressive erosion of epigenetic coherence at key regulatory regions [57].

Alteration of constitutive heterochromatin integrity has major functional consequences for genome stability. Depletion of H3K9me3-rich domains renders chromatin more vulnerable to oxidative DNA damage [90]. Moreover, aged MuSCs aberrantly recruit Polycomb complexes to γ-H2AX–marked DNA damage sites, promoting ectopic H3K27me3 deposition and further compromising transcriptional fidelity [57]. In this context, Polycomb repression becomes increasingly diffuse and mislocalized, uncoupled from its normal developmental targets.

Beyond histone modifications, aged MuSCs also display stochastic DNA methylation, increasing heterogeneity and changes in locus-specific methylation, particularly at promoter regions. Single-cell methylome and transcriptome analyses reveal that this drift is most pronounced in the dormant Pax7^Hi^ subpopulation, where stochastic promoter methylation generates transcriptional noise and disrupts coherent gene regulatory networks [91,92].

Functionally, this instability directly affects MuSC behaviour. For example, hypermethylation of the *SPRY1* promoter in aged human myogenic cells suppresses this key quiescence regulator, impairing the ability of activated MuSCs to return to quiescence. This defect accelerates depletion of the reserve stem cell pool and directly links DNA methylation changes to regenerative exhaustion [93]. These observations collectively suggest that ageing progressively erodes the stability and coherence of epigenetic states originally established during embryonic and fetal myogenesis.

Consistent with this loss of epigenetic precision, single-cell and bulk ATAC-seq analyses demonstrate that aged MuSCs fail to fully re-establish a quiescent-like chromatin architecture following regenerative cycles. Instead, they retain aberrantly open chromatin at loci normally repressed in quiescence while losing accessibility at key myogenic regulatory regions, altering MyoD occupancy at target enhancers [94]. Integrative analyses combining ATAC-seq with Hi-C further reveal age-dependent remodelling of A/B compartments and topologically associated domains (TADs). In aged MuSCs, chromatin accessibility frequently becomes uncoupled from appropriate transcriptional responses [95]. Together with the accumulation of stochastic mutations in the genome of satellite cells, these changes impair regeneration and provide a layered view of ageing in which genomic and epigenomic drift co-evolve to progressively reduce tissue robustness [96].

### (b) Metabolic rewiring amplifies epigenetic drift

Ageing progressively destabilizes the metabolic–epigenetic circuits that preserve MuSC identity and function. In aged MuSCs, dysfunction of one-carbon metabolism and disruption of SAM homeostasis impair DNA and histone methylation. As SAM flux becomes progressively diverted from histone methylation towards alternative pathways, such as polyamine synthesis, constitutive H3K9me3 heterochromatin is selectively lost, while facultative H3K27me3 domains expand. This metabolic shift disrupts heterochromatin architecture and compromises transcriptional stability, resulting in increased transcriptional noise and uncoordinated gene expression [94,97–99]. Consistent with this metabolic–epigenetic coupling, pharmacological restoration of SAM availability re-establishes heterochromatin organization, reduces DNA damage and improves regenerative capacity of aged MuSC *in vivo*, supporting a direct contribution of metabolic imbalance to functional decline in satellite cells during ageing [97,98].

Metabolic remodelling of polyamine metabolism further highlights the complexity and cell-type specificity of these changes. While metabolomic analyses of whole skeletal muscle report an approximately 50% decline in spermidine and spermine levels with age, MuSC-specific analyses reveal a paradoxical increase in spermidine levels in aged MuSCs. This difference between muscle fibres and MuSCs may reflect a compensatory response aimed at sustaining cellular MuSC functions under metabolic and epigenetic stress. However, diversion of SAM towards polyamine synthesis simultaneously limits its availability for histone methylation, thereby exacerbating heterochromatin erosion and reinforcing epigenetic drift [97,100].

Ageing is also associated with impaired OXPHOS, mitochondrial dysfunction and reduced NAD⁺ availability, collectively diminishing the supply of essential co-factors required by chromatin-modifying enzymes [80,94,97]. Reduced NAD⁺ availability limits sirtuin activity, thereby contributing to altered chromatin regulation [68,101]. Persistent mitochondrial dysfunction also promotes genotoxic stress through increased production of reactive oxygen species (ROS), causing DNA damage and altering protein function [102,103]. DNA damage signalling via γ-H2AX can in turn aberrantly recruit Polycomb complexes, potentially reinforcing repressive chromatin drift. Together, these metabolic–epigenetic perturbations correlate with increased chromatin accessibility, premature transcriptional activation and uncoordinated gene expression, contributing to maladaptive transcriptional states in aged MuSCs [57,99,102,103].

Pharmacological interventions targeting metabolic vulnerabilities can partially restore epigenetic plasticity and regenerative function. NAD⁺ precursors, such as nicotinamide riboside, enhance sirtuin activity; α-KG supplementation reactivates JmjC demethylases and one-carbon metabolites restore SAM-dependent H3K9me3 heterochromatin, increasing MuSC numbers and accelerating muscle repair in both young and aged mice [98].

Yet, inflammatory signals further destabilize aged MuSCs. Age-associated downregulation of *Kmt5a*, the enzyme responsible for H4K20me1 deposition, reduces H4K20me1 levels and sensitizes MuSCs to ferroptosis, highlighting the convergence of metabolic, inflammatory and epigenetic stressors on chromatin vulnerability [98,104–107]. Together, these findings position metabolic dysfunction not merely as a consequence of MuSC ageing but as an active driver of epigenetic noise, chromatin drift and progressive loss of regenerative competence.

### (c) Epigenetic leakage and destabilization of quiescence during muscle stem cell ageing

During ageing, MuSCs progressively lose the ability to maintain a deeply repressed and protective quiescent state. Chromatin drift, characterized by erosion of H3K9me3-enriched constitutive heterochromatin, stochastic redistribution of H3K27me3 domains and reduced robustness of INK4a/ARF repression, weakens the fidelity of transcriptional silencing required for quiescence maintenance [57,108]. As these repressive programmes loosen, aged MuSCs begin to aberrantly express stress- and senescence-associated genes even in the absence of overt damage, indicating that epigenetic noise progressively destabilizes the quiescent transcriptional landscape.

A central node in this process is the progressive derepression of the *Cdkn2a* locus. In young MuSCs, p16^Ink4a^ expression is tightly restrained by Polycomb-dependent mechanisms, including PRC1-mediated H2AK119 ubiquitination, together with transcriptional repressors, such as Slug, which directly bind the *Cdkn2a* locus to maintain its repression. With ageing, Polycomb targeting becomes less precise, and *Slug* expression declines, weakening this coordinated repression system. The resulting derepression of p16^Ink4a^ drives aged MuSCs towards a pre-senescent state characterized by impaired activation dynamics and defective maintenance of the Pax7⁺ stem cell pool [108,109].

In geriatric mice (>28 months), this instability becomes even more pronounced. MuSCs accumulate DNA damage, sustain chronic p16^Ink4a^ expression and progressively lose the capacity to maintain reversible quiescence. Activation following injury becomes delayed and unstable, and many cells prematurely exit the stem cell cycle or undergo geroconversion towards full senescence even when placed in youthful environments [110]. These pre-senescent MuSCs activate inefficiently and often irreversibly transition into a senescent state, acquiring a persistent senescence-associated secretory phenotype (SASP) which perturbs intercellular signalling and progressively degrades the regenerative niche [111,112].

Importantly, restoration of key chromatin maintenance pathways can partially reverse these defects. Reinforcing H3K9me3-dependent heterochromatin, restoring autophagy or re-establishing repression of p16^Ink4a^ improves quiescence stability and regenerative competence [102,108]. Together, these observations indicate that MuSC ageing reflects a progressive failure to maintain and properly resolve developmental repressive circuits rather than an irreversible loss of stem cell identity.

## 5. Part 3: myofibres ageing

### (a) Epigenetic ageing and multi-layered chromatin regulation of post-mitotic myofibres

#### (i) Spatial epigenetic compartmentalization in multinucleated fibres

Post-mitotic skeletal muscle fibres rely on a highly ordered epigenetic architecture to maintain fibre-type identity, metabolic specialization and NMJ integrity throughout life. Individual myofibres contain up to several thousand myonuclei that are spatially and functionally compartmentalized. Myofibres are formed through fusion of mononucleated muscle progenitors. Myofibre nuclei develop their own epigenetic identity that is strongly influenced by innervation through the NMJ and the resulting electrical activity of myofibres triggered by acetylcholine release by nerve endings. At the NMJ, a small subset of specialized postsynaptic nuclei (three to five per endplate) displays a specific chromatin organization, enriched in activating marks, such as H3K9ac and characterized by hypomethylated acetylcholine receptor promoters, thereby supporting sustained synaptic gene expression [113] (figure 2). By contrast, the vast majority of extrasynaptic nuclei (>95% of myonuclei) are enriched in heterochromatin, exhibit low levels of global H3 acetylation, repress expression of synaptic genes and mainly maintain accessibility at loci involved in metabolic and contractile functions [114–116]. This spatial organization is maintained through multiple regulatory layers, including DNA methylation patterns, histone variant turnover, post-translational modifications and non-coding RNAs. These epigenetic mechanisms are established during development and subsequently refined postnatally to support long-term myofibre homeostasis [19,117] (figure 2).

**Figure 2.**
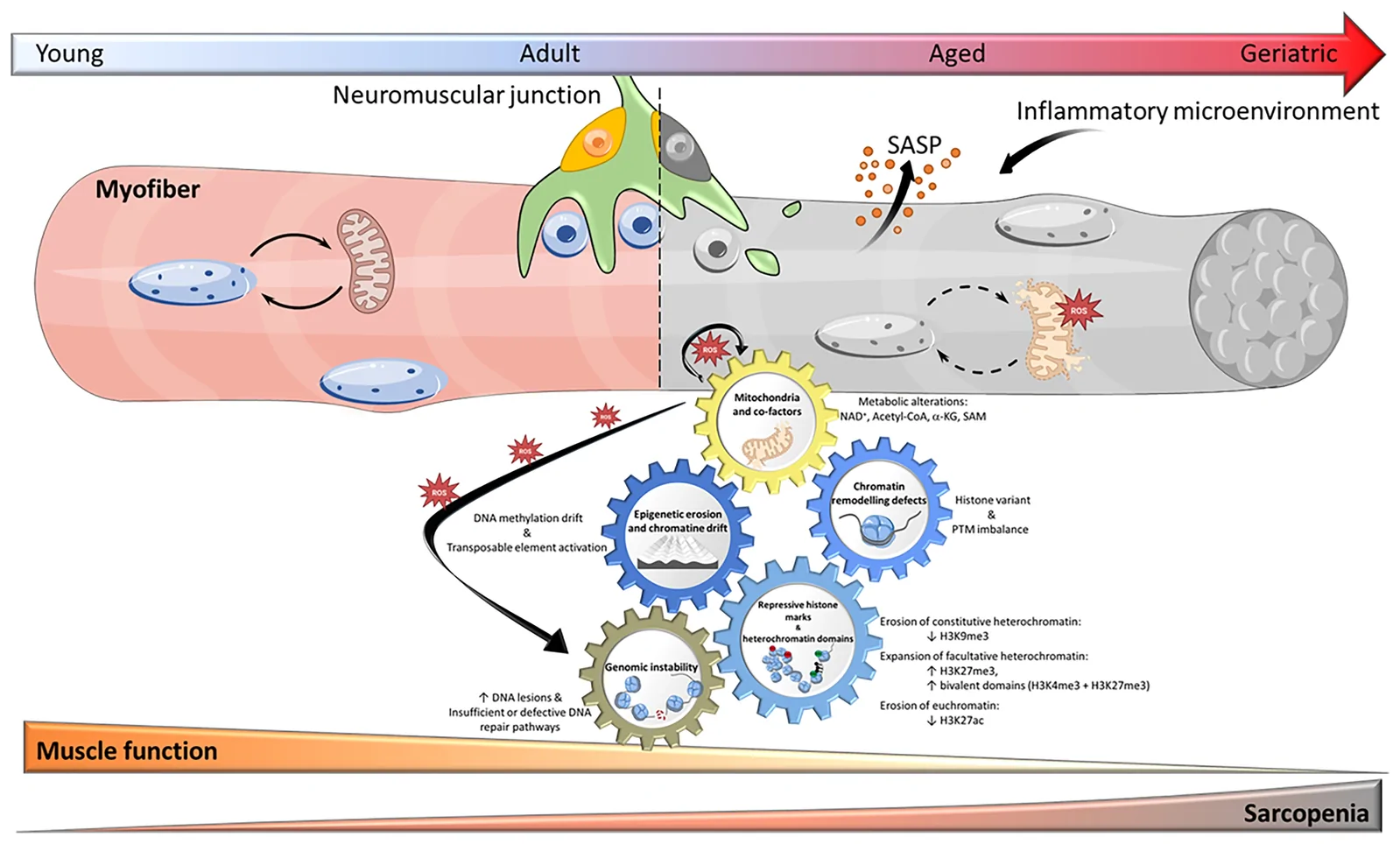
Chronological epigenetic alterations in post-mitotic myofibres during ageing. In young myofibres, chromatin organization is actively maintained, preserving transcriptional programmes required for fibre identity, NMJ integrity and contractile function. With ageing, progressive epigenetic alterations destabilize this chromatin landscape. This multi-layered chromatin dysregulation includes epigenetic erosion and DNA methylation drift, defects in chromatin remodelling and histone variant turnover and redistribution of chromatin states, characterized by erosion of constitutive heterochromatin (↓H3K9me3), expansion of facultative heterochromatin (↑H3K27me3 and bivalent domains) and erosion of euchromatin (↓H3K27ac). These chromatin alterations can further impair mitochondrial function, increasing ROS and establishing a feedback loop that amplifies cellular stress. Age-associated mitochondrial dysfunction also reduces the availability of metabolic co-factors (NAD⁺, acetyl-CoA, α-KG and SAM) required for chromatin-modifying enzymes. Together, these processes promote DNA damage and genomic instability, progressively impairing muscle function and ultimately contributing to sarcopenia.

Across the lifespan, myonuclei are exposed to cumulative stresses inherent to their post-mitotic states, including chronic metabolic fluctuations, mechanical load, oxidative stress and episodic lesions that cause local inflammation. Ageing progressively destabilizes nuclear architecture, leading to alterations in gene expression, contractile performance, mitochondrial bioenergetics, proteostasis and inflammatory signalling. Unlike proliferative cells, myofibres cannot dilute damaged chromatin or misfolded proteins through cell division. Their long-term maintenance therefore relies on replication-independent histone turnover, mitochondrial support and cell cycle-independent DNA-maintenance pathways.

Over time, these persistent stresses drive a characteristic form of epigenetic drift in ageing myofibres, which manifests by erosion of enhancer activity, impaired histone-variant dynamics, altered DNA methylation patterns and progressive accumulation of nuclear and mitochondrial DNA lesions that locally distort chromatin structure and compromise transcription-factor binding [87,118,119]. Together, these changes reduce transcriptional plasticity, rigidify chromatin organization and progressively impair metabolic flexibility and contractile homeostasis, thereby contributing to age-associated decline in muscle function (figure 2).

#### (ii) Chromatin remodelling through histone variant turnover

The remodelling and maintenance of nucleosomes in muscle fibres rely on replication-independent histone turnover. Incorporation of histone variants enables long-lived myonuclei to adapt the transcriptional landscape to contractile demands, fibre-type transitions, NMJ activity, metabolic load and environmental conditions. Among these variants, H3.3, its chaperone HIRA and the histone variant H2A.Z have emerged as central regulators of myonuclear homeostasis and ageing-associated chromatin dynamics.

*H3.3 accumulation and age-associated chromatin drift*. H3.3 progressively accumulates in ageing myofibres, representing one of the most prominent chromatin modifications in long-lived multinucleated cells. In mouse tibialis anterior muscle, H3.3 levels markedly increase between eight and 53 weeks of age and correlate with elevated H3K27me3 deposition, suggesting a shift towards a more repressive chromatin environment during ageing [120]. Functional evidence further highlights the importance of its chaperone HIRA in maintaining chromatin homeostasis. Myofibre-specific deletion of *Hira* leads to fibre hypertrophy, centralized nuclei and increased susceptibility to oxidative stress, driven by defective induction of transcriptional damage-response programmes [121]. Consistently, aged rat gastrocnemius muscle displays reduced global levels of H3K9ac, H3K27ac and H3K9me3, indicative of widespread disruption of chromatin landscape in sarcopenic muscle [122].

*H2A.Z as a guardian of myonuclear genomic stability*. H2A.Z plays a critical role in preserving genomic stability in post-mitotic myofibres. Muscle-specific deletion of H2A.Z induces oxidative stress, protein carbonylation, accumulation of DNA damage, NMJ fragmentation, mitochondrial cristae swelling, elevated ROS production and progressive muscle atrophy. These defects come along with a transcriptomic signature of accelerated muscle ageing [123]. Interestingly, young mice with muscle fibre-specific H2A.Z deficiency maintain normal transcriptional activity and regulation [124]. However, with ageing, H2A.Z becomes essential for maintaining genome integrity.

Mechanistically, H2A.Z facilitates the recruitment of the DNA repair factor Ku80 to sites of DNA damage, promoting the initiation of non-homologous end joining and limiting the accumulation of double-strand breaks (DSBs) in post-mitotic muscle fibres [123]. Loss of H2A.Z compromises efficient recruitment of DNA repair machinery at DSBs, accelerating genomic instability and structural chromatin damage in ageing myonuclei. Notably, H2A.Z deficiency also induces mitochondrial dysfunction and increased ROS production, both major sources of oxidative DNA lesions. These observations suggest the existence of a feed-forward loop linking mitochondrial dysfunction, ROS generation and chromatin instability, contributing to the progressive accumulation of genomic damage in ageing myofibres, although the precise mechanisms of this amplification remain to be fully defined.

#### (iii) DNA methylation drift and muscle epigenetic clocks

DNA methylation changes represent another major layer of epigenetic drift in ageing skeletal muscle. Early genome-wide analyses revealed that age-associated methylation changes preferentially target tissue-specific genes and often follow a predominant hypermethylation pattern [125]. The first comprehensive human methylome comparison between skeletal muscle from old (68–89 years) and younger (18–27 years) men, covering more than 480 000 CpG sites, identified 5963 differentially methylated CpGs (dmCpGs), 92% of which were hypermethylated in aged samples [126]. Age-associated dmCpGs were relatively depleted at promoter regions and were instead enriched within gene bodies, particularly in central and 3′ gene regions. Notably, intragenic methylation changes are overrepresented in genes involved in NMJ formation and maintenance [126]. From these data, over 500 CpG sites were identified to distinguish young from aged muscle [126], an approach later refined into a muscle-specific epigenetic clock [127]. Subsequent large-scale meta-analyses integrating 10–12 independent methylome datasets (*n* = 682 to 1053 human skeletal muscle samples, 18–89 years old, 22% women and 99% Caucasian) refined this muscle-specific epigenetic signature and revealed that approximately 6% of all tested CpG sites are significantly associated with chronological age in skeletal muscle [118,127]. By contrast, earlier studies suggested dominant hypermethylation; these larger datasets revealed a more balanced methylation drift, with 51–61% of differentially methylated regions (DMRs) becoming hypomethylated and 39–49% becoming hypermethylated with advancing age. Functional enrichment analyses showed that age-associated DMRs are strongly linked to pathways involved in skeletal muscle structure, development, contractile function and calcium flux regulation [118,128]. Strikingly, several genes promoting muscle atrophy become progressively hypomethylated with age, including *HDAC4* and *atrogin-1*, changes that mirror increased transcript and protein levels in ageing muscle [118]. Differential methylation also affects all *HOX* gene clusters, key regulators of developmental patterning and musculoskeletal identity, although the specific *HOX* genes and direction of methylation changes vary between studies [118,129]. At the chromatin level, Polycomb target genes and bivalent domains exhibit pronounced hypermethylation, whereas enhancers frequently display hypomethylation [118,127]. This Polycomb-associated hypermethylation is thought to reflect a gradual age-related loss of Polycomb complex protection at CpG-rich sequences, enabling progressive DNMT3-mediated methylation and thereby reducing transcriptional plasticity. Consistent with this model, hypermethylated DMRs are enriched at EZH2 binding sites and CpG islands [128,130].

Functional evidence supports the pathogenic potential of excessive *de novo* DNA methylation in muscle. Muscle-specific overexpression of Dnmt3a increases DNA methylation and induces ageing and myopathic features, including centralized myonuclei, fast-to-slow fibre-type shift, inflammation, mitochondrial OXPHOS defects, reduced basal autophagy and diminished metabolic flexibility [131].

In addition to methylation changes in genes, ageing also alters the epigenetic regulation of repetitive elements. A characteristic ‘regression towards the mean’ pattern occurs at retrotransposons, in which weakly methylated elements become hypermethylated while strongly methylated copies progressively lose methylation [132]. This drift is size-dependent, with larger retrotransposons (>5 kb) undergoing more demethylation. RNA-seq analysis further reveals a global derepression of retroelements in late ageing (20–28 months in mice), temporally coinciding with this demethylation pattern [132].

### (b) Nucleo-mitochondrial crosstalk in chromatin dynamics

Nucleo-mitochondrial communication plays a central role in shaping chromatin dynamics in muscle fibres, which must preserve epigenetic stability over decades. Mitochondrial metabolites, including NAD⁺, acetyl-CoA, SAM and α-KG, are obligate co-factors for chromatin-modifying enzymes, thereby coupling cellular metabolism to epigenetic regulation. In healthy skeletal muscle, substrate usage can flexibly shift between glucose and fatty acids depending on activity level, nutrient availability and fibre-type properties, ensuring balanced production of these metabolic epigenetic co-factors [119,133–135]. This metabolic flexibility progressively declines with ageing. Mitochondrial dysfunction, reduced NAD⁺ levels and resulting loss of sirtuin activity and increased protein acetylation and acylation become prominent, with perturbations in one-carbon metabolism and TCA cycle activity further destabilizing epigenetic homeostasis [136–138].

Declining NAD⁺ availability is particularly detrimental because it unbalances nuclear–mitochondrial communication and promotes a pseudo-hypoxic transcriptional state [133]. Beyond its role as a redox co-factor, NAD⁺ also serves as a substrate for multiple NAD^+^-dependent enzymes, including sirtuins (SIRTs), poly(ADP-ribose) polymerases (PARPs) and the NADase CD38, thereby creating competition for a shared intracellular NAD⁺ pool [139–141]. Although SIRT1 protein abundance may increase with age, its deacetylase activity nevertheless declines, reflecting limited NAD⁺ availability and increased consumption by DNA damage–responsive enzymes [138,142].

Mitochondrial dysfunction during ageing further amplifies this imbalance. Elevated levels of ROS activate PARP1 and CD38, accelerating NAD⁺ consumption. PARP1 uses NAD^+^ to synthesize poly(ADP-ribose) (PAR) chains required for DNA repair and is capable of depleting 80% of cellular NAD⁺ under oxidative stress [139,143,144]. In skeletal muscles of aged mice, both PARP1 expression and PAR levels increase, consistent with enhanced NAD⁺ flux towards PARylation [142]. Because SIRT1 and PARP1 share the same nuclear NAD⁺ pool, PARP1 activation during oxidative stress rapidly reduces NAD⁺ availability and functionally outcompetes SIRT1 even when its protein level is unchanged [72,145,146]. This imbalance becomes particularly pronounced during ageing, when cells are under chronic oxidative stress [133,147].

Paradoxically, once cells are severely depleted in NAD⁺, PARP1 itself loses its ability to sustain DNA repair, compromising the capacity of aged cells to resolve accumulating DNA lesions [148,149]. Recent work further demonstrates that PARylated PDHE1α (the alpha subunit of PDH, which participates in the NAD^+^-dependent decarboxylation of pyruvate into acetyl-CoA) can locally generate acetyl-CoA at DNA damage sites to support histone acetylation required for efficient repair [150], while PARP1-dependent nucleosome remodelling facilitates chromatin accessibility at lesion sites [151]. Although these mechanisms have not yet been directly demonstrated in skeletal muscle, they suggest that in aged myofibres PARP1 may perturb genome stability both via NAD⁺ depletion and reduced histone acetylation consequent to the loss of PDHE1α PARylation.

Acetyl-CoA metabolism provides an additional link between mitochondrial function and chromatin regulation. Nuclear acetyl-CoA production through acetyl-CoA synthetase 2 (ACSS2) complements mitochondrial supply to sustain histone acetylation. Muscle-specific deletion of *Acss2* leads to myofibre atrophy, lipid accumulation, impaired NADH regeneration and exercise intolerance [152]. These observations highlight the importance of a nucleo–mitochondrial acetyl-CoA relay in maintaining chromatin accessibility and metabolic homeostasis.

Among mitochondrial NAD⁺-dependent enzymes, SIRT3 emerges as a key regulator integrating mitochondrial metabolism with chromatin stability. In skeletal muscle, SIRT3 expression dynamically responds to exercise and coordinates downstream signalling pathways. Its induction parallels CREB phosphorylation and increased expression of the mitochondrial regulator PGC-1α. Conversely, *Sirt3* knock-out mice display reduced AMPK and CREB phosphorylation together with decreased PGC-1α expression [153]. Although primarily characterized as a mitochondrial deacetylase, emerging evidence indicates that SIRT3 may also influence nuclear chromatin organization. In human mesenchymal stem cells, SIRT3 depletion disrupts heterochromatin integrity, promotes detachment of lamina-associated domains (LADs), increases chromatin accessibility and induces aberrant repetitive element transcription, ultimately accelerating cellular senescence, whereas SIRT3 reintroduction restores chromatin organization [154].

Importantly, SIRT3 is itself embedded within stress-responsive transcriptional networks. Heat-shock factor 1 (HSF1), whose expression in skeletal muscle declines with ageing, directly activates SIRT3 transcription and promotes PGC-1α deacetylation, thereby enhancing mitochondrial function and muscle integrity while inducing fibronectin type III domain‑containing protein 5 (FNDC5)/Irisin-mediated tissue crosstalk [155]. The progressive downregulation of the HSF1–SIRT3 axis during ageing links impaired stress sensing to mitochondrial dysfunction, altered acetylation dynamics and chromatin destabilization [155]. Together, these findings support a model in which mitochondrial NAD⁺‑dependent pathways directly influence higher‑order genome organization, reinforcing the concept that nucleo‑mitochondrial coupling is a central determinant of epigenetic maintenance in ageing skeletal muscle.

## 6. Exercise-induced epigenetic plasticity and attenuation of muscle ageing

Physical activity is one of the most robust non-pharmacological interventions known to preserve skeletal muscle function during ageing. Beyond its well-established effects on proteostasis, mitochondrial function and muscle stem cell maintenance and regeneration [156–160], it has become clear that exercise is also a direct modulator of the muscle epigenome, counteracting age-related epigenetic drift across DNA methylation, histone modifications and non-coding RNAs [161,162].

Skeletal muscle displays a high epigenetic plasticity in response to exercise stimuli. Acute exercise triggers rapid chromatin remodelling and transcription factor accessibility, involving histone acetylation and DNA methylation. Early studies reported increased H3K36 acetylation at promoters of metabolically regulated genes and reduced nuclear abundance of HDAC4 and HDAC5, enhancing transcriptional elongation [163,164]. Acute endurance exercise also induces marked DNA hypomethylation in skeletal muscle of previously sedentary men and women, as well as in murine muscle tissue, in parallel with dose-dependent increases in *PGC-1α*, *PPAR-δ* and *PDK4* expression [165]. In rodents, exercise enhances histone H3 hyperacetylation at the MEF2 regulatory site and increases MEF2A binding to the *Glut4* promoter, reinforcing transcriptional activation of metabolic genes [166].

Chronic exercise also partially reverses these age-related epigenetic changes in DNA methylation. In aged mice (22–24 months), eight weeks of resistance training is sufficient to reduce promoter hypermethylation and decrease the epigenetic age of muscle by approximately 8% [13]. Human studies corroborate these findings. Resistance training remodels the muscle methylome and transcriptome in both young and older adults, partially reversing age-related changes [167]. Recurrent training cycles in older individuals partially restore molecular responsiveness to exercise, consistent with an emerging model of epigenetic muscle memory. Exercise training also offsets age-related epigenetic changes in nuclear and mitochondrial genomes, while muscle disuse accelerates ageing [168,169]. Acute resistance exercise also increases the expression of TET enzymes, responsible for DNA demethylation, without altering DNMT levels, suggesting a shift towards active epigenetic remodelling [170].

Large-scale integrative analyses further strengthen the link between physical fitness, training status and younger molecular profiles in human skeletal muscle. Using thousands of epigenetic and transcriptomic datasets across multiple cohorts, Voisin *et al.* [171] demonstrated that higher aerobic fitness is associated with younger molecular signatures, while exercise training shifts aged muscle towards a younger epigenomic and transcriptomic state. By contrast, muscle disuse or low fitness drives the opposite trajectory. These effects were particularly evident in pathways related to mitochondrial function, energy metabolism and muscle structural integrity, reinforcing the central role of mitochondrial health in muscle ageing. Importantly, these associations were not explained by differences in fibre-type composition, supporting the notion that exercise induces intrinsic remodelling of muscle regulatory programmes rather than reflecting changes in cellular composition [171]. Complementary findings indicate that lifelong physical activity is associated with promoter hypomethylation of genes involved in metabolism, myogenesis, contractile function and oxidative stress defence in aged skeletal muscle [172].

The beneficial effects of exercise on mitochondrial function and number have long been acknowledged and obviously contribute to the preservation of the epigenetic landscape during ageing. Among known metabolic/mitochondrial ameliorations provided by exercise, reduction in 8-OHdG accumulation despite increased ROS production reduces oxidative stress and DNA damage [159]. In addition, chronic endurance training increases muscle NAD⁺ availability through NAMPT induction, restoring SIRT1 activity and reducing global protein hyperacetylation in both young and aged muscle [173–175]. AMPK acts as the primary exercise sensor coordinating SIRT1-PGC-1α signalling [176], while NAMPT upregulation occurs through both AMPK-dependent and -independent pathways [177]. Chronic training further engages nuclear SIRT6, which promotes oxidative metabolism via the CREB-Sox6 axis [178].

## 7. Conclusions

Skeletal muscle ageing reflects the long-term consequences of developmental programming. Chromatin landscapes, histone variant profiles and metabolic wiring established during myogenesis confer extraordinary longevity and functional specialization to post-mitotic myofibres, but also make them vulnerable to cumulative stress. Over the lifespan, mechanical load, metabolic demand and oxidative stress cause epigenetic drift, including DNA methylation changes, histone modifications, chromatin remodelling, as well as alterations in higher-order genome architecture, such as TADs and LADs. Together, these changes progressively undermine transcriptional homeostasis, genomic stability, mitochondrial function and regenerative capacity.

This epigenetic erosion is compounded by mitochondrial dysfunction and DNA damage, creating a detrimental feedback loop that impacts not only myofibres but also satellite cells, the microenvironment, NMJs and systemic myokine signalling.

Importantly, exercise and metabolic interventions can preserve and partially restore the chromatin landscape and nucleo-mitochondrial communication during ageing, highlighting the importance of exercise to delay sarcopenia.

Understanding the interplay between developmental programming, epigenetic drift and metabolic regulation reframes muscle ageing as a lifespan disorder rather than mere wear-and-tear, and opens new avenues for interventions aimed at preserving epigenetic youth and functional longevity in skeletal muscle.

## Data Availability

This article has no additional data.

## Theme

One contribution of 21 to a theme issue ‘Developmental circuits in ageing’.
